# Case of a Variety of Intermittent Fever, &c.

**Published:** 1803-12-01

**Authors:** C. L. Dumas

**Affiliations:** Member of the National Institute, President of the Society, &c.


CASE *of a* VARIETY *of* INTERMITTENT FEVER, &c. *by* C. L. DUMAS, *Member of the National Institute, President of the Society, &c.*

[ Concluded from p. 430. ]

7th. THE patient appeared sufficiently calm this day. The author thought proper to reduce the dose of bark; two drachms only were ordered at five o'clock in the morning, and a drachm every six hours, until the moment of the paroxysm on the ensuing day, and which came on much more early than was expected, being at least two hours sooner than the period of the preceding paroxysm. The only bad symptoms remarkable in this fit were those of hydrophobia, the others were hardly observable. In the intervals of calm that occured between the symptoms already described, the patient seemed to enjoy his mental faculties completely; the horror of water only remained, and for which he had an antipathy that no reasoning could shake. The access finished later, and consequently continued longer than the last.

The 8th. M. Dumas profited of this day's intermission to push the bark in larger doses, and resumed his first quantity of half an ounce at once, and a drachm every six hours. This day was remarkable only for a copious sweat, and a continued tendency to drowsiness.

On the 9th, the hour of the paroxysm was not remarkable for the slightest degree of fever, deglutition only became more difficult; aversion for liquids was perceivable; in the night a slight degree of fever made its appearance, with some delirium, but unaccompanied by any effort to bite, &c. The access was become almost null or at least much moderated in comparison to the preceding, and it was terminated by abundant sweats and quiet sleep.

The following day, Sept. 10th, the patient was extremely low, having hardly force sufficient to stand upright; the desire of sleep was unceasing, and he complained of great thirst, but no disposition whatever to eat. He was ordered to continue the bark, but at greater intervals, and in smaller doses; two drachms were taken morning and night, and in the day the dose was reduced to a drachm. During the three or four following days there was neither fever, delirium, nor hydrophopbia; a convalescent state was manifest, and the patient was recommended to continue for some time longer the use of the bark.

